# Mitochondrial Functionality in Inflammatory Pathology-Modulatory Role of Physical Activity

**DOI:** 10.3390/life11010061

**Published:** 2021-01-15

**Authors:** Rafael A. Casuso, Jesús R. Huertas

**Affiliations:** Department of Physiology, Institute of Nutrition and Food Technology, University of Granada, 18100 Granada, Spain

**Keywords:** exercise, mitochondria, immune system, metabolic disease, COVID-19

## Abstract

The incidence and severity of metabolic diseases can be reduced by introducing healthy lifestyle habits including moderate exercise. A common observation in age-related metabolic diseases is an increment in systemic inflammation (the so-called inflammaging) where mitochondrial reactive oxygen species (ROS) production may have a key role. Exercise prevents these metabolic pathologies, at least in part, due to its ability to alter immunometabolism, e.g., reducing systemic inflammation and by improving immune cell metabolism. Here, we review how exercise regulates immunometabolism within contracting muscles. In fact, we discuss how circulating and resident macrophages alter their function due to mitochondrial signaling, and we propose how these effects can be triggered within skeletal muscle in response to exercise. Finally, we also describe how exercise-induced mitochondrial adaptations can help to fight against virus infection. Moreover, the fact that moderate exercise increases circulating immune cells must be taken into account by public health agencies, as it may help prevent virus spread. This is of interest in order to face not only acute respiratory-related coronavirus (SARS-CoV) responsible for the COVID-19 pandemic but also for future virus infection challenges.

## 1. Introduction

Immunometabolism is a recently proposed term which highlight the close relationship between systemic and cellular metabolism and the immune system [1]. On one hand, immunometabolism studies show how the metabolism of immune cells ultimately regulates their function [2]. On the other hand, they also cover the chronic activation of the immune system that is observed in several pathologies such as obesity, Type 2 Diabetes, cardiovascular diseases and cancer [1,2]. However, these two events should not be viewed as independent processes. In fact, mitochondrial signaling from bone marrow cells stimulates macrophage polarization and their infiltration into tissues thereby controlling diet-inducing obesity [3]. In fact, mitochondrial metabolism has a key role in regulating both innate immune cell function during infection and metabolic disease development (for detailed reviews, see [4,5]).

In the present review, we describe how exercise regulates immune function, reduces systemic inflammation and ultimately results in improving age-related metabolic diseases. We also highlight a close relationship between the mitochondrial function of immune cells and their functional infiltration into tissues thereby altering systemic metabolism. Finally, we propose a scenario where exercise improves skeletal muscle macrophage–mitochondria crosstalk, which will likely enhance skeletal muscle metabolism and prevent age-related skeletal muscle dysfunction.

## 2. Exercise Alters Systemic Inflammatory State: Role of Exercise Intensity

The immune system responds to exercise following a hormetic curve. Notably, an immunological threshold has been recently proposed for exercise intensity (up to 60% of oxygen uptake) and duration (up to 60 min) [6]. In this scenario of moderate exercise intensity and/or duration (i.e., moderate exercise), an acute exercise bout increase the exchange of leukocytes between circulation and tissues; in addition, anti-inflammatory cytokines are released and tissue macrophages show antipathogen activity [7,8]. Moreover, the fact that natural killer cells and CD38+ T lymphocytes enrich the blood compartment [7,9] has been used to state that acute moderate exercise may protect from subsequent infection [6]. Therefore, this kind of exercise exerts a number of physiological responses that may act as a barrier for acute virus infection and maybe its spread.

In contrast, high-volume vigorous exercise (i.e., vigorous exercise) is associated with up to six-fold upper respiratory tract infections in the weeks following it. This effect has been associated with increments in circulating lymphocytes and neutrophils [10] probably due to a decreased levels of NK activity as well as T cell and macrophage function [11,12]. Importantly, this effect not only reflects a high metabolic stress but can also be triggered by skeletal muscle damage leading to pro-inflammatory cytokine release [13] and a strong innate immune response [14,15]. Notably, Casuso et al. asked highly trained subjects in both swimming and running to perform 8 bouts of 30 s at maximal intensity (~30 min) one day by swimming and one day by running. It was reported that circulating levels of interleukin 6 (IL-6) raised 2 h after running but not after swimming. Moreover, this effect was not due to muscle damage nor to metabolic stress (i.e., cortisol release), and a general anti-inflammatory response was observed after both exercise types [16]. This suggests that (i) immune response to exercise may differ between exercise modes and/or due to the contracting muscle and that (ii) extremely intense exercise does not perturb the immune response in trained subjects, at least if there is no excessive duration.

The chronic application of moderate-intensity exercise bouts (i.e., training) is known to decrease illness and lower systemic inflammation [6,17]. It is less clear how regular vigorous exercise affects the immune system. Nevertheless, it is tempting to suggest that the acute depression of the immune system described above would follow a homeostatic response thereby showing an enhanced effect in the long term. What is clear is that regular moderate exercise can prevent systemic inflammation.

## 3. Exercise Impact Chronic Inflammation in Age and Age-Related Metabolic Diseases

Many chronic diseases are associated with persistent low-grade systemic inflammation. For instance, immune cells can infiltrate in adipose tissue thereby promoting a pro-inflammatory environment leading to insulin resistance and Type 2 Diabetes [18,19]. Moreover, a similar polarization and infiltration of the immune cells has been observed in cardiovascular diseases and in some cancers [20,21,22]. Franceschi et al. (2018) proposed that aging and ageing-related metabolic diseases should be studied as a whole as they converge in systemic inflammation (i.e., inflammaging) and both directly and indirectly influence each other. In fact, they propose that metabolic inflammation driven, for example, by nutrient excess and inflammaging probably share the same molecular pathways [23]. Although this remains to be empirically demonstrated, it seems evident that age and age-related metabolic diseases show increased levels of pro-inflammatory cytokines [24]. This is likely due to a decrease in the body´s ability to repair damaged cells or tissues, thus leading to accumulated damage over time. In fact, a chronic activation of the innate immune system underlies the inflammaging process where macrophages have a key role [25].

In this scenario, twenty years ago, Pedersen´s laboratory first described that contracting muscle release IL-6 into the circulation indeed altered the function of other parts of the body [26]. For instance, muscle-derived IL-6 is known to reduce systemic inflammation by promoting cortisol and IL-10 release [27,28]; this is of importance because IL-10 blocks the transcription of the inflammatory cytokine tumor necrosis factor apha (TNF-α) [29]. It is now known that skeletal muscle secretes hundreds of peptides (i.e., myokines), most of them with unknown effects. While the main studied one remains IL-6, other myokines have been described to affect bone, liver, adipose tissue, brain and muscle function, among others (see Sverinsen and Pedersen [30] for a detailed updated review). Nevertheless, it is important to note that IL-6 has a double-edged effect depending on the secreting cell. In contrast to exercise, chronic sedentarism polarizes macrophages, thereby releasing pro-inflammatory cytokines such as IL-6, IL-1b and TNFα [31].

Exercise has been recommended as a therapy for a number of metabolic diseases such as obesity, type 2 diabetes as well as for cardiovascular diseases and cancer [32]. Notably, most of these diseases are directly or indirectly affected by contracting muscle-derived IL-6. For instance, IL-6 receptor blockade impedes exercise-induced visceral and cardiac adipose tissue reduction [33,34]. Additionally, IL-6 regulates glucose uptake by promoting glucose transporter 4 (GLUT4) translocation to the sarcolemma [35].

Moreover, sarcopenia (i.e., age-related muscle and/or force decline) has also been associated with increments in circulating levels of IL-6 and TNF-α [36]. Studies in mice showed that genetic loss of IL-6 impair skeletal muscle hypertrophy [37]. While these observations seem to contrast, it can be explained because, as noted above, chronic muscle disuse promotes IL-6 release from macrophages, thereby inducing a pro-inflammatory environment.

Nevertheless, few studies have addressed macrophage infiltration in response to exercise. Walton et al. [38] reported that 14 weeks of resistance training increases anti-inflammatory macrophage content within aged human skeletal muscle. Moreover, studies analyzing cycling training show that anti-inflammatory macrophages may have a key role in stimulating muscle growth [39]. More recently, Jensen et al. [40] analyzed the time course of macrophage infiltration and polarization following acute resistance exercise. They found that within the range of 4 to 7 days after exercise, older males show an increase in anti-inflammatory macrophages, while older females show an increase in both anti- and pro-inflammatory macrophages [40]. Notably, while young females show unchanged macrophage polarization and infiltration, 5 days following exercise, there is an increase in total macrophage and anti-inflammatory cytokines in older compared to young females [40]. Taken together, these data suggest that macrophage polarization influences circulating pro- and anti-inflammatory cytokines which may directly or indirectly impact age-related muscle waste. Moreover, there is a need to unravel how resistance exercise-induced alterations in macrophage polarization influence sarcopenia and the potential role (if any) of sexual dimorphism. 

## 4. The Mitochondria–Macrophage Connection Regulates Metabolism

Mitochondria are double membrane organelles that support cellular function including metabolism and signaling. The inner mitochondrial membrane contains the electron transport chain (ETC), a multiprotein complex system that pumps protons from the mitochondrial matrix into the intermembrane; complex V can use the stored potential energy to generate ATP. This highlights the importance of mitochondria in integrating fuel metabolism to cellular ATP production. However, mitochondria are now widely recognized as biosynthetic hubs such as for nucleotide synthesis, fatty acid and cholesterol synthesis, amino acid synthesis, and glucose and heme synthesis, but they also orchestrate waste management [41]. Therefore, mitochondria are contemporarily viewed as a multifaceted organelle. Of particular relevance is the fact that mitochondrial reactive oxygen species (ROS) production acts as a signaling molecule that ultimately results (if produced in excess) in the increased inflammation observed in obesity and obesity-related diseases [42]. In this regard, excessive ROS production by ETC complex I was observed when it was not assembled with complex III and/or complex IV forming supercomplexes [43,44]. In agreement, skeletal muscle of diabetic subjects showed a decrease in the content of supercomplexes [45]. Therefore, ETC supramolecular organization may contribute to regulating metabolic diseases by controlling ROS production. However, the precise role of mitochondrial ROS production in health and disease is yet to be elucidated. For instance, it seems that mitochondrial ROS production via reverse electron transport improves lifespan in model organisms [46]. This may suggest that “when” and “where” mitochondrial ROS are produced are relevant factors in order to switch from beneficial to adverse adaptations. 

**Figure 1:**
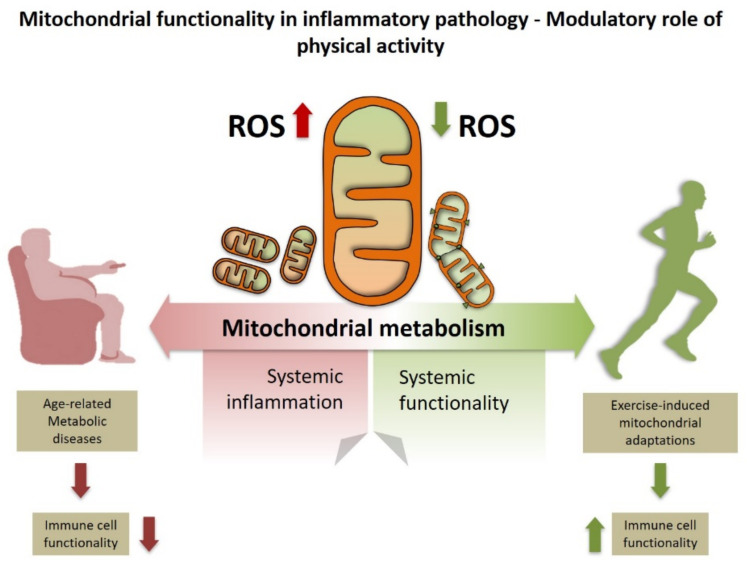
Exercise improves systemic inflammation by enhancing mitochondrial metabolism. This effect likely involves decreased mitochondrial reactive oxygen species (ROS) production below pathological levels which may ultimately affect immune cell function.

As stated above, immunometabolism can refer either to (i) how immune cells function depending on their own metabolism or to (ii) how chronic systemic inflammation influences the development and progression of metabolic diseases. However, these two apparently different processes might be influenced by each other. For instance, infiltration of pro-inflammatory macrophages into adipose tissue drives metabolic dysfunction through the expression of TNF-a, IL-1B and IL-6 [47]. Notably, excessive ROS production leads to Fgr activation in macrophages which is associated with increased mitochondrial complex II activity and complex I degradation leading to pro-inflammatory macrophage polarization [48,49]. Recently, Acín-Perez et al. [3] effectively demonstrated that mice lacking Fgr are protected against high fat diet-induced obesity and insulin resistance. Furthermore, transplanting bone marrow cells from mice overexpressing mitochondrial catalase protects mice from high fat diet-induced obesity and changes tissue macrophage towards an anti-inflammatory polarization. Therefore these data not only suggest that bone-marrow-derived cell metabolism might influence obesity and insulin resistance but also that prevention of excessive mitochondrial H_2_O_2_ production may be an interesting target for the prevention and treatment of metabolic disease. Moreover, it also opens the possibility that local mitochondrial ROS production has a key role in regulating systemic inflammation and metabolic disease. In this regard, it is important to note that moderate exercise training gradually decreases ROS production within contracting muscle [50]. This ultimately maintains ROS production at physiological levels, thus avoiding pathological ROS production. In addition, we recently reported that moderate exercise training changes the supramolecular organization of the ETC, which prevents excessive mitochondrial ROS production and systemic lipid peroxidation [51]. These findings open new interesting hypotheses such as whether exercise training alters mitochondrial function from circulating and/or skeletal muscle resident macrophages, thereby improving overall health (Figure 1).

Additionally, these observations could also anticipate that exercise-induced skeletal muscle mitochondrial metabolic adaptations result in enhancing resident macrophage function. In this regard, a second recent discovery shows that cardiomyocytes release subcellular particles called exophers which mainly transport defective mitochondria. These exophers are captured and eliminated by cardiac macrophages, thus maintaining cardiomyocyte homeostasis [52]. Notably, the authors identified Mertk as the macrophage phagocytic receptor of exophers [52]. This study has a profound impact not only on understanding the metabolic regulation of cardiac cells, but it also may help to unravel how skeletal muscle responds to stress. Runyan et al. [53] analyzed skeletal muscle macrophages from old and young mice during recovery from influenza A infection-induced pneumonia. They found that macrophages from old mice had a reduced phagocytic function which coincided with reduced Merk expression, thus leading to impaired muscle recovery. Moreover, when they knocked down Mertk in young mice, they found that muscle recovery was also impaired [53]. Cardiomyocytes have evolved to allow continuous beating by maintaining mitochondrial homeostasis through ejecting dysfunctional mitochondrial portions [52]. Given the similar structure between skeletal muscle and cardiac cells and that Mertk might have an impact on skeletal muscle recovery following infection, it would be important to study whether highly trained skeletal muscle may also have such a macrophage function as mitochondrial waste management. In fact, highly trained skeletal muscle shows a highly specialized mitochondrial network which facilitates energy diffusion through skeletal muscle cells [54]. Notably, the capacity to reach this adaptation is almost intact in aged human skeletal muscle [55]. This is of importance as muscle regenerative capacity is impaired in aged skeletal muscle [56]. Therefore, aged skeletal muscle cells show a low renewal rate but almost intact mitochondrial function (at least in response to exercise) which somewhat mimics the physiology of cardiomyocytes [57]. These recent studies open a new line of investigation of age-related skeletal muscle dysfunction.

Finally, it is important to highlight another key finding that might have an important implication in understanding how skeletal muscle mitochondria connect immunity and metabolism in mammals. There is growing interest in elucidating how contracting muscles use lipid droplets (LD) in order to sustain exercise metabolism and/or for unknown purposes. For instance, during high-volume, high-intensity exercise (i.e., 57 min and 11 mmol/L blood lactate), LD within myofibrils but not those LD located close to the sarcolemma are used [58]. This is surprising as this kind of exercise mostly relies on oxidative metabolism mainly through fatty acid and glucose oxidation [59]. LDs have important implications in cellular innate defense and, in hepatocytes, this effect seems to be dependent upon LD–mitochondria binding [60]. In fact, pathogen-associated molecular pattern lipopolysaccharide (LPS) stimulation-induced innate immune response was prevented when perilipin-5 (PLN5) was overexpressed [60]. Notably, both in sedentary and trained skeletal muscle, moderate exercise seems to decrease LD–PLN5 association [61]. As PLN5 is involved in mitochondria–LD tethering, these observations could suggest that moderate exercise liberates LD from mitochondria, thus impeding any competition between mitochondria and bacteria for LD tethering. This further supports the observation that acute moderate exercise prevents subsequent infection in this case within skeletal muscle. Furthermore, during innate immune response, LD increase their size [62]. This may also reveal different effects of LDs within skeletal muscle fibers, for instance, type 2 fibers have lower LD density, but they seems to have higher size than those LD form type 1 fibers in the leg skeletal muscle [58]. Although the current evidence is scarce, it would be interesting to test whether exercise impacts innate immune function by altering LD–mitochondria contact.

## 5. Mitochondria at the Crossroad to Viral Infection

Although it is not the primary aim of the present review, given the actual situation due to the COVID-19 pandemic, we would like to discuss how skeletal muscle mitochondrial fitness may help to face virus infection. It has been reported in a cohort of 249 subjects (59 ± 12 years old) that maximal exercise capacity was independently and inversely associated with the likelihood for hospitalization due to COVID-19 [63]. This is in accordance with the fact that obesity is a risk factor for increasing severity due to virus infection and that obesity impairs antibody response to the influenza vaccine [64]. Therefore, general fitness is a protective factor to virus infection severity in general and COVID-19 disease in particular.

Damage-associated molecular patterns are the pathway by which innate immune cells recognize pathogens leading to specific immune response. A growing body of evidence highlights the key role of mitochondria, in addition to the ROS-mediated mechanisms, in innate immunity. The reader is referred to some excellent new reviews on the topic [65,66], because we will not describe in detail these molecular mechanisms. In brief, when viruses reach the cytoplasm, RNAs perceived as foreign activate the aggregation of an outer mitochondrial membrane protein called mitochondrial antiviral-signaling protein (MAVs). MAV aggregation induces the expression factor kappa light chain enhancer of activated B cell (NF-kB) and type I interferons via interferon regulatory factors (IRFs) in the nucleus, thereby leading to antiviral defense [65,66].

However, this pathway alters mitochondrial metabolism and morphology. It has been reported that mitochondrial dynamics (i.e., balance between mitochondrial fusion and fission) and mitophagy seem to be important to the regulation of immune response to virus infection [67]. In fact, some viruses are able to alter the innate immune system in cells by increasing DRP1 s616 phosphorylation [68], which ultimately results in mitochondrial fragmentation and dysfunction [66]. Additionally, peripheral mononuclear cells showed altered mitochondrial metabolism by SARS-CoV-2 in patients with COVID-19 [69]. Moreover, the authors proposed that disease severity was positively associated with the degree of mitochondrial dysfunction in peripheral mononuclear cells [69].

These observations are of great interest in order to describe how exercise can hamper virus severity and function. For instance, sedentary aged skeletal muscle shows an enlarged and dysfunctional mitochondrial network due to impaired fission and mitophagy [55]. This may contribute to the increased risk of older subjects to show a higher severity COVID-19 disease. In this regard, moderate exercise is able to improve skeletal muscle mitochondrial function and dynamics [54,70] even in aged skeletal muscle [55]. In particular, only 12 weeks of moderate exercise can decrease skeletal muscle DRP1 s616 phosphorylation, thereby enhancing mitochondrial network function in older subjects [71]. Furthermore, short-term exercise training improves the mitochondrial function and dynamics of peripheral blood mononuclear cells even in previously trained subjects [72]. Therefore, it is tempting to suggest that moderate exercise inducing skeletal muscle and systemic mitochondrial adaptations may lower the degree of virus severity and complications related to COVID-19.

## 6. Conclusions

**Figure 2:**
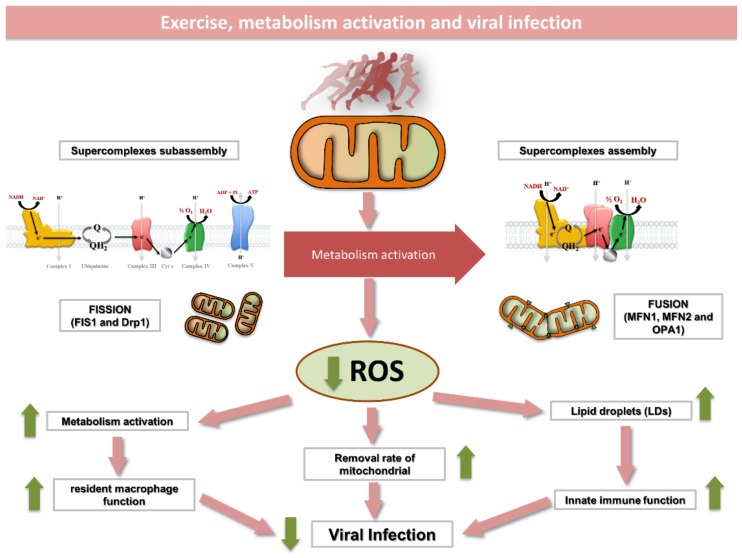
Exercise induces a number of structural and metabolic changes within skeletal muscle mitochondria that may affect immune function. Exercise enhances mitochondrial function by (1) inducing the assembly of respiratory complexes into supercomplexes; (2) promoting an enlargement of functional mitochondrial network; (3) increasing mitochondrial turnover and (4) reducing mitochondrial ROS production. The immunological role of skeletal muscle lipid droplets and resident macrophages in response to exercise is yet to be elucidated. Mechanistic studies, however, suggest that LDs separated from mitochondria can improve innate immune function in a mechanism likely mimicked by exercise. In addition, resident macrophages are known to improve mitochondrial function through eliminating dysfunctional parts of the mitochondrial network. As some viruses alter the innate immune system in cells by inducing excessive mitochondrial fragmentation, exercise may help to face virus infection by maintaining an enlarged and fully functional mitochondrial network. ROS, reactive oxygen species; DRP, dynamin-like protein; FIS1, fission, mitochondrial 1; MFN, mitofusin; OPA1, optic atrophy 1.

Several age-associated pathologies show increased systemic inflammatory markers. These metabolic diseases are worsened if a sedentary state is maintained for a long period, and in this process, the pro-inflammatory polarization of tissue resident macrophages seems to have a key role. In contrast, exercise stimulates a raising number of known myokines which target several tissues and protect them against metabolic diseases. In this scenario, we suggest that acute exercise might polarize circulating macrophages through limiting mitochondrial ROS production, thereby reducing tissue metabolic dysregulation. Moreover, we propose that both skeletal muscle macrophages and LDs may have an important role in the improvement of innate immunity observed in response to training in a process fine-tuned by mitochondria (Figure 2). Finally, we would like to highlight that acute moderate exercise training likely reduces virus infection through increasing circulating lymphocytes and leukocytes and by improving mitochondrial function. This must be acknowledged by public health agencies in order to face the current COVID-19 disease and future virus-related pandemics.

## Data Availability

No new data were created or analyzed in this study. Data sharing is not applicable to this article.
